# Optimizing the Radiology Experience through Radiologist–Patient Interaction

**DOI:** 10.7759/cureus.8172

**Published:** 2020-05-17

**Authors:** Andrew W Phillips, Rebecca A Landon, Gregory S Stacy, Larry Dixon, Andrea L Magee, Stephen D Thomas, Xi Dai, Christopher Straus

**Affiliations:** 1 Emergency Medicine, University of North Carolina, Chapel Hill, USA; 2 Radiology, The University of Chicago Medicine, Chicago, USA; 3 Nuclear Medicine, Thoracic Imaging, The University of Chicago Medicine, Chicago, USA

**Keywords:** radiology, patient education, mri knee

## Abstract

Objective

The goal of this survey-based study is to explore patients’ knowledge of and expectations for radiologists in the outpatient setting.

Materials and Methods

A comprehensive survey was distributed to adult patients undergoing knee magnetic resonance imaging (MRI) over a one-year period from September 2015 through August 2016 at an urban, quaternary care academic medical center.

Results

The survey results demonstrate that only a subset of patients undergoing knee MRI at the institution during the survey period are aware of the role of the radiologist, which is a well-documented fact described in the literature. Approximately one-third of patients expected to meet the radiologist during their visit to the department of radiology to undergo a knee MRI. The vast majority of patients surveyed wanted to be able to contact the person who read their exam, but only one patient actually contacted the radiologist during the study period.

Conclusion

While the vast majority of surveyed patients wanted to be able to contact the person who read their knee MRI, only one patient actually did reach out to the radiologist to discuss findings. However, six of 36 follow-up respondents reported that they had contacted the person “who interpreted/read your exam:” two in person, one by email, three by phone, and one by other. Survey results demonstrated that only a subset of patients correctly understood the role of the radiologist (46% in the 1^st ^survey and 63% in the 2^nd ^survey, which does not represent a statistically significant difference), which suggests that perhaps the patients did have a conversation with a member of the radiology department staff whom they believed was actually the radiologist. The fact that patients expressed a desire to communicate with the person reading their reports, but then did not take advantage of the opportunity to contact the radiologist, suggests that the issue is more complicated than just a lack of a pathway for communication between patients and radiologists. Perhaps the lack of a clear understanding of the role of the radiologist hinders patients from contacting radiologists, as they feel uncertain as to whom they are actually attempting to reach. Or perhaps patients are sufficiently reassured by having a means through which they could contact the radiologist and do not require the actual communication in order to feel comfortable. There remains a significant amount of work to be done in understanding the barriers in patient-radiologist communications.

## Introduction

Reporting practices in radiology represent a critical component of the patient’s medical record in both the inpatient and outpatient settings. There has been considerable interest within the radiology literature to evaluate the efficiency of radiology reporting from the standpoint of the referring physician, patient, and radiologist [1-4]. Specific attention has been given to the matter of how and by whom results are communicated to the patient, with the recent emphasis on autonomy and patient centeredness. 

Despite the advent of electronic medical records and implementation of patient portals, patients remain generally dissatisfied with how they receive their medical imaging results [1-4]. Patients want access to relatively detailed information with minimal delay [5-7]. Recent literature demonstrates that patients prefer to receive their imaging results directly from the referring physician and would like both the report and the original images to be available to them [6,8-9]. 

Public awareness of the radiologist’s role in their care, however, is limited, with only a subset of patients correctly identifying the role of the radiologist in several recent surveys addressing this issue [7,10]. The lack of awareness of the radiologist’s role in these studies is understandable considering that radiologists often operate outside of direct patient interaction. Some studies, however, have shown that patients felt positive toward meeting the radiologist interpreting their examination, and this is the patient attitude that this study sought to explore [10]. Thus, this study sought to evaluate a simple communication intervention to improve patients’ understanding of the radiologist’s role and their satisfaction with the medical imaging communication process.

## Materials and methods

Study population

This study was approved by the University of Chicago’s institutional review board (IRB) as an IRB exemption and was HIPAA compliant. All adult patients over the age of 18 undergoing knee MRI during the data collection period from September 1, 2015 through August 16, 2016 were eligible. Patients without a known email address were excluded since this was a primary means of communication. Please see Appendix A for a sample communication.

Survey design

The survey was designed and implemented by the radiology department staff and a statistician. Four basic areas were assessed through the survey: 1) patient demographic information, 2) patient knowledge of radiology including perception of the radiologist’s role, 3) patient opinions on methods for communication of test results, and 4) patients’ perceived ease of access to their own health care record. Please see Appendix B for the complete survey instrument.

Survey protocol

Patients were contacted to obtain consent at least one business day prior to their scheduled MRI appointment. Following consent, a pre-appointment survey was administered by email. The pre-appointment survey restated that the subject’s participation was voluntary, could be terminated at any time, and would have no effect on clinical care. To incentivize participation, entry into a drawing for a small prize was offered to patients upon completion of the survey. 

**Figure 1:**
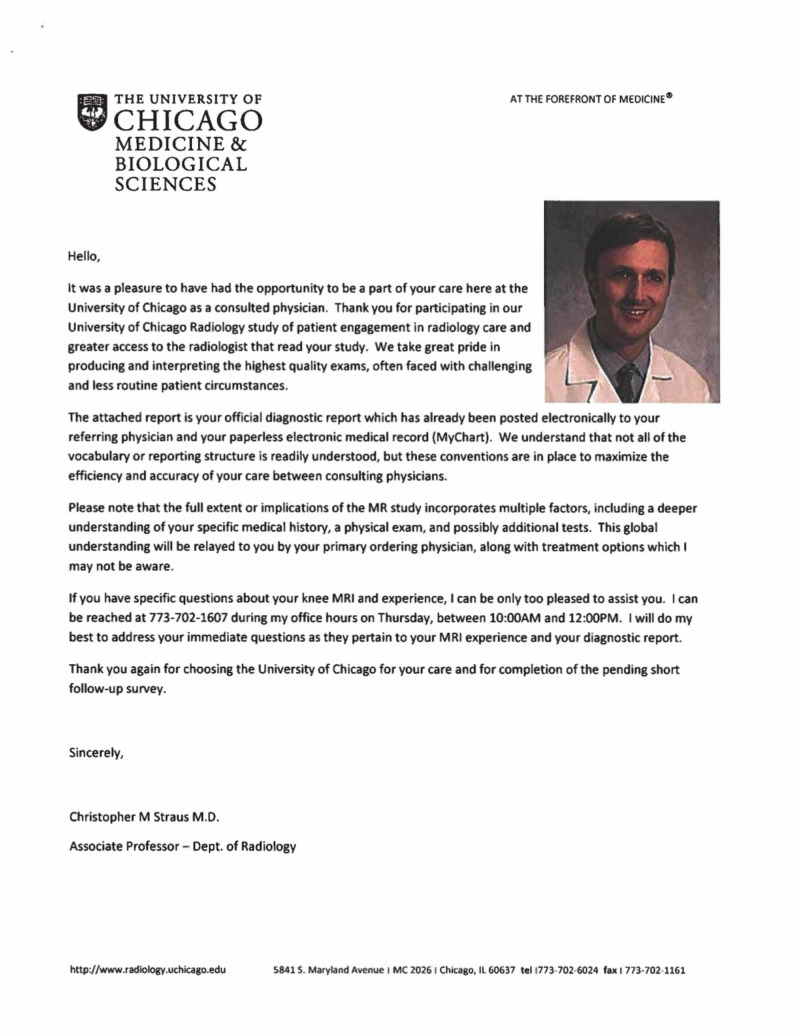
Cover letter

The participating patients underwent the scheduled knee MRI as per the standard hospital radiology department protocol. MRI knee protocol comprises a group of MRI sequences to routinely assess the internal pathology of the knee. Within two to four business days following the MRI, participating patients received a copy of the diagnostic report with the intervention: a cover letter introducing the attending radiologist who interpreted the MRI (including a photograph, greeting from the radiologist and invitation to communicate directly with the radiologist about the interpretation). Please see Figure 1 of Appendices for the complete cover letter. In this written communication, patients were encouraged to contact the radiologist with any questions or comments regarding the imaging study or the overall experience in the radiology department. A second post-appointment survey was administered to patients two weeks after the date of the MRI. Collected survey data and exchanges with the radiologists were processed through a departmental data broker (Human Imaging Research Office (HIRO)) for the diagnostic reports. 

Statistical analysis

Data were collected via REDCap (REDCap Consortium, Nashville, TN) and analyzed in SPSS v24 (IBM Corp, Armonk, NY). Descriptive statistics and univariate analyses were used as indicated. Chi-square was used for nominal comparisons and independent t-tests for continuous variable comparison. Nonresponse bias for the surveys was evaluated with a population comparison against age and sex of all knee MRIs performed during the study period [11]. 

## Results

One hundred thirty-five patients were contacted, of whom, 63 declined to participate, three were excluded for not having an email address, and 69 consented to participate. Forty-nine consented participants responded at least partially to the first survey, and 36 consented participants responded at least partially to the follow-up survey, yielding a response rate of 25.6% (AAPOR response rate definition #4). Four musculoskeletal fellowship-trained attending radiologists participated. Three of the 135 potential respondents who were contacted did not have an email address and were excluded. Using the same percentage, we estimated that 2.3% of potential respondents who did not respond would also be ineligible. This is accounted for in the response rate calculation per the AAPOR guidelines [11].

Demographics

The majority of the 49 respondents were female (71.4%), and the mean (standard deviation) age was 42.4 (14.6). Most (95.9%) of the 49 respondents had prior medical imaging, and 71.4% had a prior MRI. Almost a third of the total (49) respondents reported highest education obtained to be a graduate degree (30.6%), 20.4% a bachelor’s degree, 40.8% an associate’s degree or “some college,” and 8.2% a high school degree or equivalent.

**Table 1:** Respondent demographics SD, standard deviation; GED, graduate equivalent degree; MRI, magnetic resonance imaging

Characteristic	n (%) or n ± SD
Age	42.4 ± 14.6
Female	35 (71.4)
Had any prior imaging	47 (95.9)
Had prior MRI	35 (71.4)
Highest education obtained	
High school or GED	4 (8.2)
Some college	17 (34.7)
Associate’s degree	3 (6.1)
Bachelor’s degree	10 (20.4)
Graduate degree	15 (30.6)

Role of the radiologist

Fewer than half (47.9%) of 48 respondents initially reported that a radiologist is a “medical doctor.” After receiving the cover letter with information about the radiologist and an invitation to communicate with him or her (the intervention), 69.4% of 36 respondents reported that they thought a radiologist was a “medical doctor,” which was not significantly different from before the intervention, regardless of whether or not “don’t know” responses were included (*Χ*^2^(2)=4.233, *p*=0.120) and (*Χ*^2^(1)=2.451, *p*=0.117), respectively.

No statistically significant differences were observed in patients’ understanding of the radiologist’s role after the intervention. Of 49 pre-intervention respondents, 77.6% reported that the radiologist “interpret[s] the images,” 44.9% reported that the radiologist “run[s] the machine that takes the images,” and 18.4% reported that the radiologist "maintain[s] the machine that takes the images.” Of 36 follow-up respondents, 77.8% reported that the radiologist interpreted the images (*Χ*^2^(1)=0.001, *p*=0.980); 30.6% reported that the radiologist ran the machine (*Χ*^2^(1)=1.797, *p*=0.180); and 8.3% reported that the radiologist maintained the machines (*Χ*^2^(1)=1.723, *p*=0.189).

Approximately 1/3 (31.3%) of 48 respondents expected to meet the radiologist during the knee MRI, while 37.5 % did not, and 31.3% were unsure. Twenty-two per cent (22.2%) of the 36 follow-up respondents reported that they met the radiologist during their knee MRI, and 2.8% (one patient) were unsure.

**Table 2:** Pre- and post-intervention understanding of radiologists’ role in patient care

Radiologist Role or Characteristic	Pre-Intervention	Post-Intervention	P-value
Radiologist run[s] the machine that takes the images.			
Yes	22 (44.9)	11 (30.6)	.180
No	27 (55.1)	25 (69.4)	
Radiologist interpret[s] the images.			
Yes	38 (77.6)	28 (77.8)	.980
No	11 (22.4)	8 (22.2)	
Radiologist maintain[s] the machine that takes the images.			
Yes	9 (18.4)	3 (8.3)	.189
No	40 (81.6)	33 (91.7)	
Radiologist is a medical doctor.			
Yes	23 (47.9)	25 (69.4)	.120
No	18 (37.5)	9 (25.0)	
I don’t know	7 (14.6)	2 (5.6)	

Imaging experience 

The vast majority (75.5%) of the 49 respondents expected to have their imaging results within two days after the exam (22.4% same day, 24.5% one day after, 28.6% two days after). 10.2% expected results in one week; the few remaining respondents were spread between three and five days.

After the intervention, most (83.3%) of the 36 follow-up respondents expected future outpatient medical imaging results by two days after the exam (11.1% same day, 27.8% one day after, 44.4% two days after), *Χ*^2^(30)=75.663, *p*<0.001 for all response options).

Communication with the radiologist

The vast majority (85.4%) of 48 respondents wanted to be able to contact the person who read their exam, with a preference for email (51% of 49 respondents) and MyChart® (44.9% of 49 respondents). In-person and phone communication (32.7% and 36.7%, both of 49 respondents) were less preferred. One patient wanted to communicate with the person reading the imaging “through the ordering doctor.” MyChart®is the patient-facing component of the institution’s electronic health record through which patients can communicate with physicians.

Only one patient called the radiologist and discussed results for 12 minutes. However, six of 36 follow-up respondents reported that they had contacted the person “who interpreted/read your exam:” two in person, one by email, three by phone, and one by “other” but did not specify (one person used two methods of contact).

Nonresponse bias analysis

The study's demographic evaluation found no evidence of nonresponse bias based on age (Mann-Whitney U test for non-normal data, *p*=0.297) and gender (*Χ*^2^(1)=1.070, *p*=0.308).

Validity and reliability analysis

The study opted to evaluate validity with Cronbach’s more traditional measures because of the binomial and cognitive nature of our questionnaires. Content validity was carried out through evaluation by a survey expert (AP) in addition to several attending radiologists who all had more than ten years of experience. The dichotomous data did not lend itself to a Cronbach’s alpha for internal consistency analysis; however, several expected relationships existed that support the construct validity. Specifically, those who thought that a radiologist runs the machine that takes the images were more likely to expect to meet the radiologist (*Χ*^2^(2)=20.028, *p*<0.001) in the pre-appointment questionnaire and report that they did meet the radiologist during the exam (*Χ*^2^(2)=15.818, *p*<0.001) in the post-appointment questionnaire. Additionally, respondents who answered that a radiologist interpreted the images were more likely to also respond that the radiologist is a physician in both the pre (*Χ*^2^(2)=7.602, *p*=0.022) and post (*Χ*^2^(2)=17.589, *p*<0.001) appointment questionnaires.

## Discussion

Our study found that many patients, despite being a highly educated cohort, did not understand radiologists’ role in their care, a finding that did not improve significantly after a personal introductory communication from the radiologist. We also found discordance between patients’ expressed desire to communicate with their radiologist and their actual rate of personal communication with their radiologist.

The first-round survey results re-demonstrated several current trends in the radiology literature. First, concordant with the recent trends toward patient autonomy and medical record access, patients desire access to their medical records--including radiology reports--and desire short turnaround intervals between completion of a radiologic examination and production of a report. Interestingly, although the vast majority of participants felt that it was desirable for the physician interpreting the examination to be available for discussion, only one out of 49 participants actually did engage with the radiologist. This suggests that perhaps the availability of the radiologist is reassuring in that it provides patients with an option, but ultimately patients are generally satisfied with the potential for the encounter with the radiologist without actually needing to engage in it. This is an area in which further exploration is needed.

Public awareness of the role of the radiologist is limited, with only a subset of patients correctly identifying the role of the radiologist in recent surveys addressing this issue [7,10]. In our pre-intervention survey, only 23% of respondents reported that the radiologist is a medical doctor. Interestingly, when the level of education was compared to responses to this question, an inverse correlation between education and knowing that radiologists are physicians was found. Simply put: the more educated a patient was, the less likely s/he was to know that a radiologist is a physician. This lack of understanding regarding the role of the radiologist may contribute to limited patient enthusiasm for consultation with the radiologists who read their imaging in our study. The knowledge gap is likely present because radiologists tend to operate outside of direct patient interaction. However, it is not clear from our intervention or survey why increasing levels of education are associated with the misunderstanding. 

Although the vast majority of surveyed patients wanted to be able to contact the person who read their knee MRI, only one patient actually did reach out to the radiologist to discuss findings. Interestingly, six of 36 follow-up respondents reported that they had contacted the person “who interpreted/read your exam:” two in person, one by email, three by phone, and one by unspecified “other.” It is likely that these patients communicated with the ordering physician, thinking s/he interpreted the image since that has historically been the patient's source for imaging results. The number also aligns relatively well with the approximately 25% of our patients who did not think the radiologist was the person interpreting the imaging. Alternatively, the patients are mistaking other radiology staff for the radiologist. Technicians should be reminded to properly introduce themselves and their role to ensure patients’ understanding.

The fact that patients expressed a desire to communicate with the person reading their reports, but then did not take advantage of the opportunity to contact the radiologist, suggests that the issue is more complicated than just a lack of a pathway for communication between patients and radiologists. Perhaps the lack of clear understanding of the role of the radiologist hinders patients from contacting radiologists since they feel uncertain as to whom they are actually attempting to reach. Or perhaps patients are sufficiently reassured by just having a means through which they could contact the radiologist, and do not require the actual communication in order to feel comfortable.

Limitations

This study was limited by its single-institution design and relatively small sample size; the follow-up survey had a relatively low response rate as well. Additionally, patients were only screened for inclusion criteria three days a week, and many scans were conducted in less than 24 hours, excluding them from participation. It is also possible that there would be different results with a different imaging modality or body region. 

## Conclusions

Since a personalized communication to patients for a specific exam was unsuccessful in educating patients, ultimately informing patients about the vital role that radiologists contribute to their care and encouraging patients to make radiologists an active member of their healthcare team may be very difficult in the current healthcare model and should concern the radiology community. Future research should delve into why patients are not actually communicating with their radiologist when given the opportunity and explore working with colleagues in other specialities to help the radiology community inform patients of their care role.

## Figures and Tables

**Figure 2:**
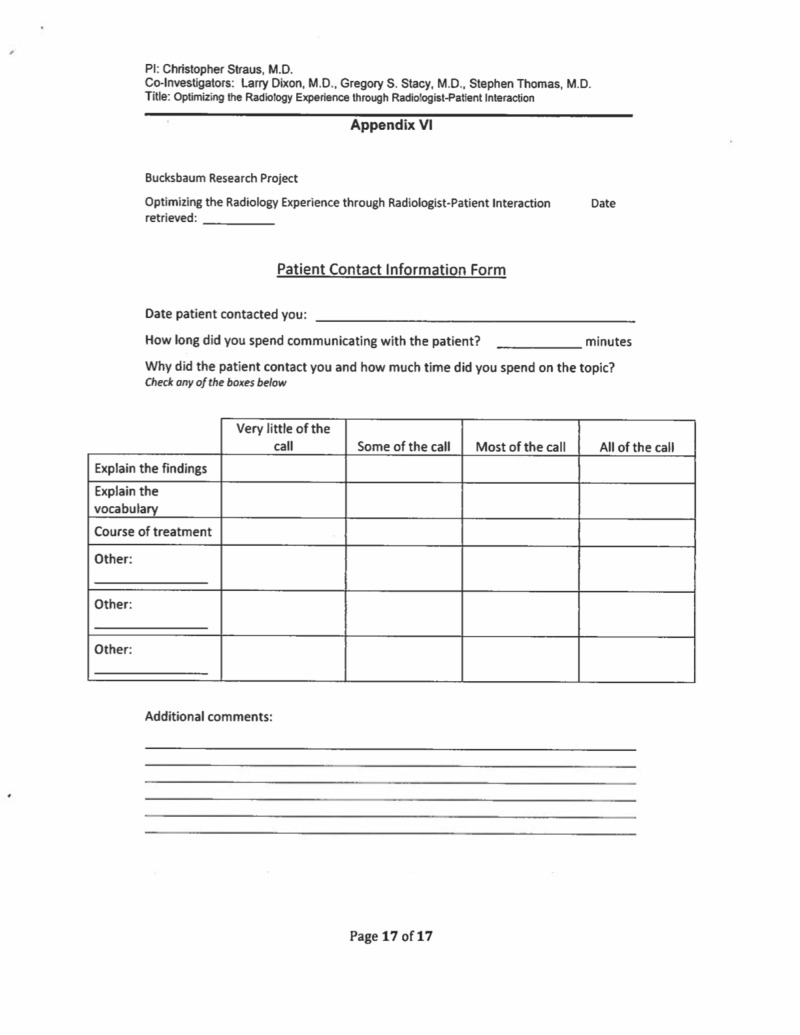
Patient contact information form

**Figure 3:**
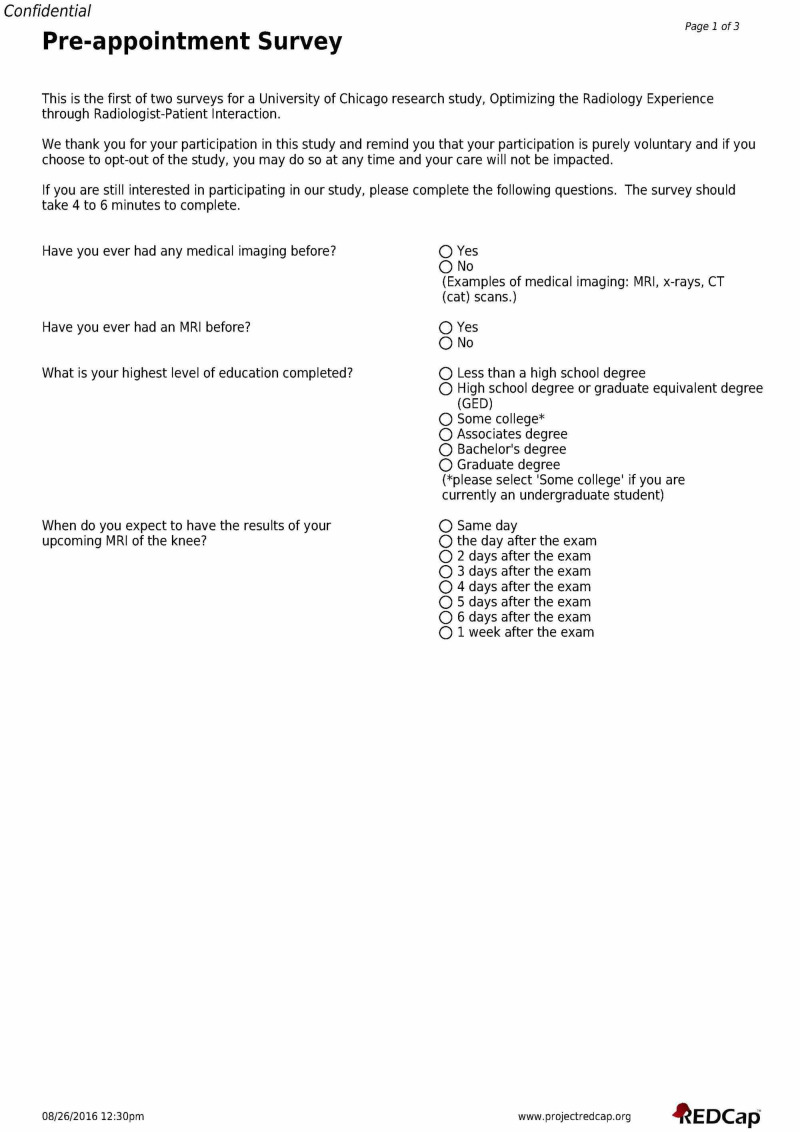
Pre-appointment survey 1 of 3

**Figure 4:**
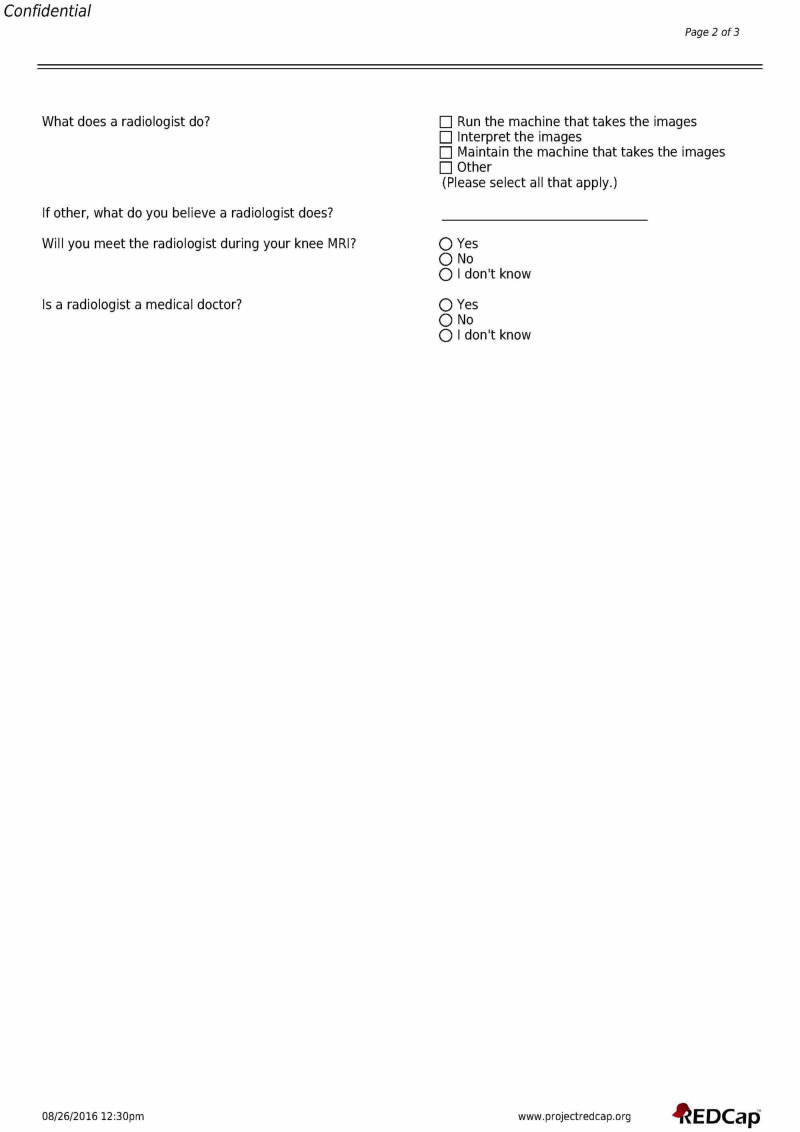
Pre-appointment survey 2 of 3

**Figure 5:**
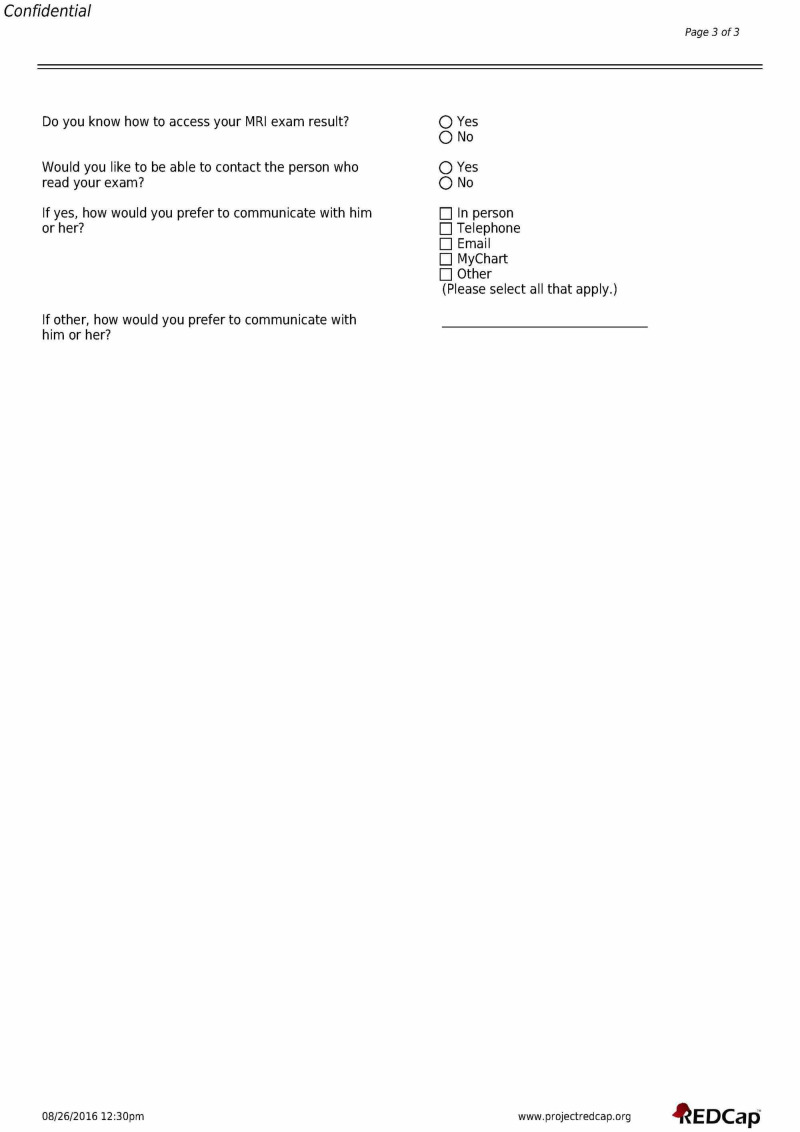
Pre-appointment survey 3 of 3

**Figure 6:**
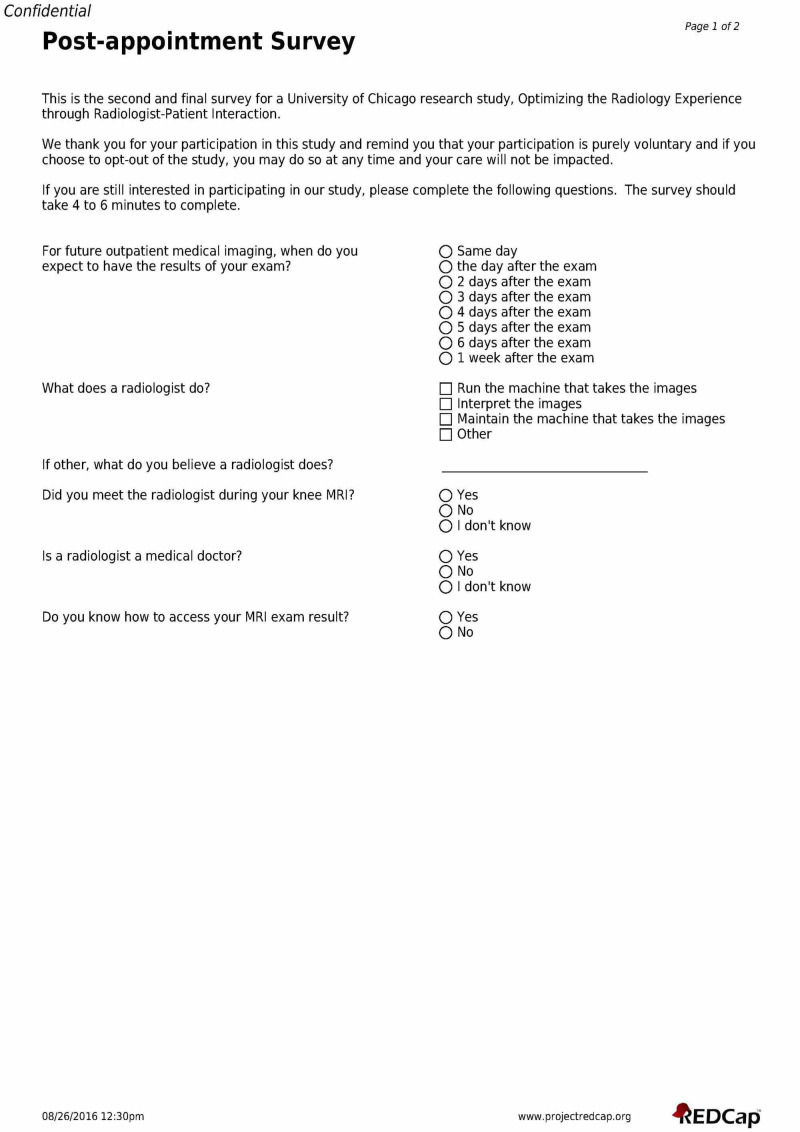
Post-appointment survey 1 of 2

**Figure 7:**
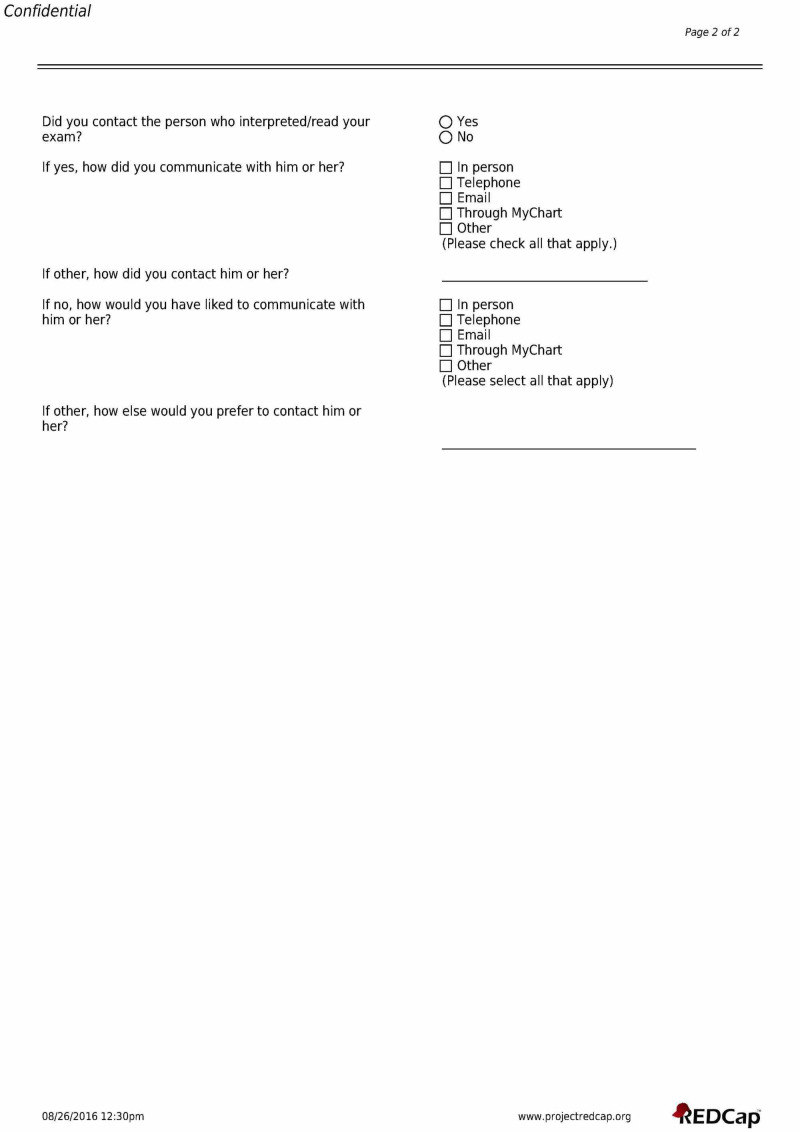
Post-appointment survey 2 of 2
